# Efficacy and Safety of Intravenous-to-Oral Lascufloxacin Switch Therapy in Community-Onset Pneumonia: A Single-Arm, Open-Label Clinical Trial

**DOI:** 10.7759/cureus.80404

**Published:** 2025-03-11

**Authors:** Naoki Iwanaga, Naoki Hosogaya, Takahiro Takazono, Yusei Tsukamoto, Ryosuke Morio, Satoshi Irifune, Takuto Miyamura, Yosuke Harada, Yohsuke Nagayoshi, Akira Kondo, Tomo Mihara, Yoshihisa Kohno, Yuichi Fukuda, Tsutomu Kobayashi, Eisuke Sasaki, Toyomitsu Sawai, Yoshifumi Imamura, Toru Morikawa, Kohji Hashiguchi, Yoji Futsuki, Yuichi Inoue, Kiyoyasu Fukushima, Naofumi Suyama, Hiroaki Senju, Hikaru Tanaka, Yurika Kawazoe, Shimpei Morimoto, Yuya Ito, Masataka Yoshida, Kazuaki Takeda, Shotaro Ide, Noriho Sakamoto, Koichi Izumikawa, Katsunori Yanagihara, Hiroshi Mukae

**Affiliations:** 1 Department of Respiratory Medicine, Nagasaki University Hospital, Nagasaki, JPN; 2 Clinical Research Center, Nagasaki University Hospital, Nagasaki, JPN; 3 Department of Infectious Diseases, Nagasaki University Graduate School of Biomedical Sciences, Nagasaki, JPN; 4 Department of Internal Medicine, Izumikawa Hospital, Minamishimabara, JPN; 5 Department of Respiratory Medicine, Japanese Red Cross Nagasaki Genbaku Hospital, Nagasaki, JPN; 6 Department of Internal Medicine, Nagasaki Prefecture Shimabara Hospital, Shimabara, JPN; 7 Department of Respiratory Medicine, Sasebo City General Hospital, Sasebo, JPN; 8 Department of Internal Medicine, Saiseikai Nagasaki Hospital, Nagasaki, JPN; 9 Department of Respiratory Medicine, Japanese Red Cross Nagasaki Genbaku Isahaya Hospital, Isahaya, JPN; 10 Department of Respiratory Medicine, National Hospital Organization (NHO) Nagasaki Medical Center, Nagasaki, JPN; 11 Department of Respiratory Medicine, Japan Community Healthcare Organization Isahaya General Hospital, Isahaya, JPN; 12 Department of Internal Medicine, Kouseikai Hospital, Nagasaki, JPN; 13 Department of Respiratory Medicine, Sasebo Chuo Hospital, Sasebo, JPN; 14 Department of Respiratory Medicine, Ureshino Medical Center, Ureshino, JPN; 15 Department of Respiratory Medicine, Nagasaki Harbor Medical Center, Nagasaki, JPN; 16 Department of Internal Medicine, Nagasaki Memorial Hospital, Nagasaki, JPN; 17 Department of Internal Medicine, Aino Memorial Hospital, Unzen, JPN; 18 Department of Internal Medicine, Senju Hospital, Sasebo, JPN; 19 Infectious Diseases Experts Training Center, Nagasaki University Hospital, Nagasaki, JPN; 20 Department of Laboratory Medicine, Nagasaki University Hospital, Nagasaki, JPN

**Keywords:** community-acquired pneumonia, community-onset pneumonia, lascufloxacin, non-randomized clinical trial, switch therapy

## Abstract

Background and objective: For treating community-acquired pneumonia (CAP) in adults, early switching from injectable to oral antimicrobials (switch therapy) is accepted once the clinical course is favorable. Lascufloxacin (LSFX) is a quinolone antibacterial agent, available in intravenous and oral formulations, demonstrating antibacterial activity against a relatively broad spectrum of community-onset pneumonia (COP). No switch therapy using the same drug from injectable to oral antimicrobials has been reported; therefore, we conducted the study to confirm the efficacy and safety of the switch therapy using LSFX.

Method: We conducted an open-label, uncontrolled, multicenter study across 16 hospitals from April 2023 to February 2024 to evaluate the efficacy and safety of LSFX switch therapy against mild-to-moderate COP. Once the switch criteria were fulfilled on days 3-5, switch therapy was initiated. The primary endpoint was the cure rate at the time of test of cure (TOC). Secondary endpoints included the proportion of patients receiving switch therapy, clinical efficacy at the end of treatment (EOT), early clinical response, microbiological response at the EOT, and adverse events. The adverse events were collected from the population for the safety analysis set.

Results: The median age of the participants was 73 years, and the overall switch therapy implementation rate was 114/120 (95%), aligned with approximately 99/104 (95%) of the switch therapy performed by day three after initiating the therapy. The cure or effective rate was 100/104 (96.2%, 95% confidence interval (CI): 90.44-98.94) at TOC, 101/104 (97.1%, 95% CI: 91.80-99.40) at the early clinical efficacy testing, and 103/104 (99.0%, 95% CI: 94.76-99.98) at EOT. Adverse events related to the study drug were reported in 10.0% of the patients, with hepatic dysfunction as the most common adverse effect. Severe LSFX-induced adverse events were not observed, excluding worsening pneumonia.

Conclusion: Switch therapy using LSFX presented high efficacy and acceptable safety profiles against mild-to-moderate severity of COP. This strategy of using the same drug in both intravenous and oral formulations is quite innovative. LSFX may potentially emerge as one of the preferred options for treating COP.

## Introduction

According to the Ministry of Health, Labor, and Welfare's Vital Statistics, pneumonia was the fifth leading cause of death in Japan in 2022. In addition, a combination of pneumonia with aspiration pneumonia is the third leading cause of death. A possible cause of the rising mortality rate of pneumonia in Japan may be due to the rapidly aging population; age is directly associated with poor prognosis. According to the Ministry of Health, Labor, and Welfare’s Vital Statistics, in 2012, more than 98% of pneumonia-related deaths occurred in individuals aged 65 years or older. Based on where it is acquired, pneumonia is categorized into community-acquired pneumonia (CAP), nursing and healthcare-associated pneumonia (NHCAP), and hospital-acquired pneumonia. NHCAP is unique to Japan and includes the following criteria: admission to a long-term care or medical facility, discharge from hospital within the previous 90 days, elderly or disabled patients who require long-term care, and outpatients who frequently receive infusion treatment (including dialysis, antimicrobials, chemotherapy, and immunosuppressive drugs).

Older adults comprise most cases of aspiration pneumonia [1]. A study that prospectively enrolled patients with CAP and NHCAP examined 1,772 community-onset pneumonia (COP) cases and showed that aspiration was involved in 38.2% of all COP, 25.4% of CAP, and 64.3% of NHCAP [2]. This finding suggests that the aspiration pneumonia burden in COP may be more significant than previously reported. 16S rRNA gene analysis performed to comprehensively evaluate bacterial flora in CAP patients revealed that oral *Streptococcus* spp., previously not considered pathogenic owing to their commensal nature, are commonly found in mixed infections with anaerobes that are difficult to isolate through sputum culture [3,4]. Moreover, as the risk of aspiration increased, oral *Streptococcus* spp. was most frequently detected in CAP, indicating that oral *Streptococcus* spp. may play a pathogenic role in aspiration pneumonia associated with CAP [5]. Therefore, given that most pneumonia cases in the elderly may be aspiration pneumonia and anti-*Pseudomonas aeruginosa* (*P. aeruginosa*) agents are often not recommended for COP treatment in the elderly, antimicrobial agents targeting the oral microbiota are necessary. Owing to the progressive cognitive decline and frailty worsening during hospitalization, the length of hospitalization for pneumonia treatment in the elderly should be as short as possible.

Lascufloxacin (LSFX) has shown stronger antibacterial activity than preexisting quinolones against oral *Streptococcus* spp. and anaerobic bacteria in vitro [6,7]. While preexisting quinolones strongly inhibit either DNA gyrase, which is involved in bacterial DNA replication, or topoisomerase IV, which is involved in the separation of double strands after DNA replication, LSFX inhibits both DNA gyrase and topoisomerase IV, preventing the development of resistant strains [8]. Moreover, LSFX exhibits antibacterial activity against preexisting quinolone-resistant strains [9]. In a Japanese phase III clinical trial, the efficacy of oral LSFX tablets and levofloxacin for CAP treatment was comparable. In addition, we reported that LSFX tablets are safe and effective in the treatment of moderate NHCAP [10], suggesting that LSFX represents a promising therapy for aspiration pneumonia in elderly patients.

Switch therapy involves switching from intravenous to oral antibiotic administration, contributes to shorter hospital stays, lowers healthcare costs [11-13], and improves patient satisfaction without treatment failure or worse prognosis in many cases of CAP [14]. Switching from penicillins or cephalosporins to preexisting new quinolones has been reported [15,16]. Although switching to a different class of drugs carried a relative risk of adverse effects, no studies performing the switch therapy using the same drug have been reported. Therefore, in this study, we aimed to determine the efficacy and safety of LSFX switch therapy in treating COP by conducting an open-label, uncontrolled, multicenter study across 16 Nagasaki University-affiliated hospitals.

## Materials and methods

Study design

This clinical trial was a physician-initiated, open-label, uncontrolled, multicenter study. This clinical trial was approved by the Clinical Research Review Board of Nagasaki University (approval number: CRB22-005) and was registered in the Japan Registry of Clinical Trials (study number: jRCTs 071230001). The study was conducted in compliance with the Clinical Research Act and the World Medical Association Code of Ethics on Experiments on Human Subjects (Declaration of Helsinki). Written informed consent was obtained from each patient before enrollment into the study. The study was conducted at the following 16 facilities: Nagasaki University Hospital, Japanese Red Cross Nagasaki Genbaku Hospital, Nagasaki Harbor Medical Center, Nagasaki Saiseikai Hospital, Japanese Red Cross Nagasaki Genbaku Isahaya Hospital, Japan Community Healthcare Organization Isahaya General Hospital, Japanese Aino Memorial Hospital, Nagasaki Kouseikai Hospital, National Hospital Organization (NHO) Nagasaki Medical Center, Sasebo City General Medical Center, Sasebo Chuo Hospital, Ureshino Medical Center, Senju Hospital, Izumikawa Hospital, Nagasaki Memorial Hospital, and Shimabara Hospital.

Inclusion and exclusion criteria

Adults over 18 years old with at least one of the following: cough, purulent sputum or worsening sputum purity, abnormal findings on auscultation or percussion (including crackles, dull percussion, and decreased breath sounds), dyspnea or tachypnea, fever (axillary temperature ≥ 37°C), leukocytosis (>10,000/mm^3^) or rod-shaped nucleated cells > 15%, positive C-reactive protein (CRP), or hypoxemia (partial pressure of arterial oxygen (PaO_2_) ≤ 60 Torr or saturation of percutaneous oxygen (SpO_2_) ≤ 90%) were included in the study. In addition, only patients with an acutely apparent infiltrative shadow on chest radiography or chest CT images 48 hours prior to LSFX administration were included in the study. Patients with mild or moderate disease, according to the A-DROP (age, dehydration, respiration, disorientation, and blood pressure) system recommended by the Japanese Respiratory Society, were included [17]. The main exclusion criteria were a history of hypersensitivity to quinolone antimicrobial agents, systemic administration of other antimicrobial agents seven days before study drug administration, QTc prolongation (QTc ≥ 500 ms), hypokalemia (K < 3.5 mEq/L), severe hepatic dysfunction (Child-Pugh classification Grade C), and Class IA or Class III antiarrhythmic drugs. The detailed exclusion criteria are presented in Appendix A.

Interventions

Patients with COP were intravenously administered 300 mg of LSFX injection on the first day of treatment and were injected with 150 mg of LSFX injection on the second day of treatment, following the drug reference. Improvement or no worsening of clinical symptoms (cough, sputum, and dyspnea) since the start of treatment, ability to take oral medication, and no fever (38.0°C or higher) for at least 12 hours were adopted for the switch criteria. One of the original reports on switch therapy showed that in a prospective cohort observational study of 200 consecutive admissions for CAP, 133 patients (67%) were switched to oral antibiotics within three days of admission, and clinical failure was observed in only one patient [14], suggesting that the switch timeframe looked reasonable around the third day during the hospital stay. In addition, referring to previous reports with switch therapy on days three [13], four [12,16], and 3-5 [18], the timing of the switch therapy in this study was determined to be on days 3-5. Once the switch criteria were met from the third to the fifth day, the LSFX tablet (75 mg) was orally administered once daily for a total of around seven consecutive days for the injection and tablets. If prolonged therapy was deemed necessary by the attending physician, it was administered for up to 14 days. Early clinical response was assessed on day three (+2 days allowed, shown in Appendix B) and at the end of treatment (EOT) on day seven (+3 days allowed, shown in Appendix B). Test of cure (TOC) was examined and observed one week after the EOT (five to 10 days after the EOT, as shown in Appendix C). Patients were allowed to leave the hospital when they met the discharge criteria: switch therapy, improvement in respiratory status, and discharge at the physician’s discretion. If the principal investigator or subprincipal investigator advised that the research drug be discontinued owing to an adverse event, or if patients or their proxies request to withdraw from the study, the study drug was immediately discontinued. The details of the prohibited medications are provided in Appendix D.

Study endpoints

The primary endpoint was the cure rate at TOC among the study subjects who received switch therapy. Secondary endpoints were (1) the proportion of patients who switched study drugs, (2) the cure rates or effective rates at early clinical evaluation and EOT, (3) the length of hospital stay, and (4) the factors associated with the use of switch therapy. As an exploratory analysis, factors associated with the achievement of cure rates at TOC, defined as the primary endpoint, were identified, before and after treatment initiation, in an exploratory manner. The analysis population was stratified by causative organism, age, A-DROP score, and presence or absence of complications, and the cure rate at TOC was analyzed. Adverse events were collected from the population for the safety analysis set (SAS), which was defined as patients who were enrolled as study participants and received at least one dose of the study drug during the study period.

Microbiological assessments

The sputum obtained prior to drug administration and at the time of TOC determination was sent to Kyurin Corporation (Yahatanishi-ku, Kitakyushu City, Japan) for culture testing. After culturing, the materials were sent to the Hibiki AMR Research Group, where the minimum growth-inhibitory concentrations of various antibiotics, including LSFX, were determined. Based on the quantitative sputum culture, the physician-in-charge determined the pneumonia-causing organism. Sera obtained immediately before study drug administration and at the time of TOC were sent to the SRL Corporation (Shinjuku, Tokyo, Japan), where *Mycoplasma* antibodies and *Chlamydia pneumoniae* IgA and IgG were measured and compared. If the increase ratio of each antibody at TOC, compared to before treatment, was more than four times, the test was regarded as positive. In addition, *Legionella* urinary antigen levels were measured before drug administration.

Statistical analysis

The target population for the SAS was defined as patients who were enrolled as study participants and received at least one dose of the study drug during the study period. The target population for the full analysis set (FAS) was defined as the subset of SAS consisting of patients who could be assessed as either “cured” or “not cured” at TOC. The modified FAS (mFAS) was defined as an extension of the FAS that included patients who could be defined as “cured” or “not cured” at TOC or withdrew from protocol treatment. The target population for the per-protocol analysis set (PPS) was defined as patients with at least 80% adherence to the protocol treatment (study drug administration). The objective of the primary analysis was to determine the lower bound of the 95% confidence interval (CI) for the binomial probability of “cure” frequency, using the Clopper-Pearson method with FAS or PPS as the target population. The target number of patients was calculated based on the CI of the binomial distribution for the primary endpoint (cure rate at TOC): assuming a cure rate of 95.2% for LSFX-switched patients, an alpha error of 5%, and a probability of achieving accuracy (1-β) of 80%, the minimum sample size was determined such that the upper and lower bounds of the 95% CI for the cure rate were 10% or less. The minimum sample size that resulted in a lower bound of 10% or less was 97 (Wilson score interval). Furthermore, assuming that 10% of the incorporated cases would be “undecidable,” switch therapy would be implemented in 108 cases. Assuming a switch rate of 90% from injection to oral administration, injections would be administered in 120 cases.

The sensitivity analysis was designed to assess upward bias in estimating the probability of treatment success, resulting from the exclusion of cases that withdrew from the protocol therapy before the TOC assessment was performed. For this purpose, the FAS was the target population for sensitivity analysis, and the Clopper-Pearson method was used to calculate 95% CIs for the binomial probabilities. For the analysis of secondary endpoints, 95% CIs of binomial probabilities were obtained as in the primary analysis; however, the target population was set in the FAS.

## Results

**Figure 1:**
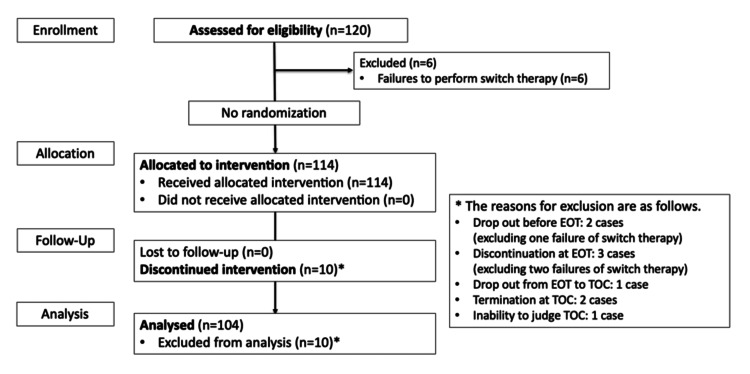
The diagram of patient disposition During the study period, 120 patients provided written informed consent, and six patients who did not meet the criteria for switching from infusion to tablets were excluded. Then, 10 patients who discontinued TOC for other reasons were excluded. EOT: end of treatment; TOC: test of cure

The patient flow in this study is shown in Figure 1.

During the study period, 120 patients provided written informed consent and were included in the SAS population for safety evaluation. Six patients who did not meet the criteria for switching from LSFX infusion to LSFX tablets were excluded from the analysis. Overall, switch therapy was feasible in 114 (95%) of 120 patients. Next, 10 patients who discontinued TOC for other reasons were excluded; 104 patients were finally included in the FAS population, the primary population for evaluating clinical efficacy (Figure 1). The characteristics of the FAS population are summarized in Table 1.

**Table 1:** Baseline characteristics (FAS) IQR: interquartile range; CAP: community-acquired pneumonia; FAS: full analysis set; NHCAP: nursing and healthcare-associated pneumonia; COP: community-onset pneumonia; A-DROP: age, dehydration, respiration, disorientation, and blood pressure; BUN: blood urea nitrogen; SpO_2_; saturation of percutaneous oxygen; PaO_2_; partial pressure of arterial oxygen A-DROP score: 0 means mild, 1 or 2 means moderate, 3 means severe, and 4 or 5 means extremely severe.

Characteristics	Objective	N = 104
Age (years)	Median (IQR)	72 (62-79)
Sex	Male (%)/female (%)	61 (58.7)/43 (41.3)
CAP	Yes (%)	90 (86.5)
NHCAP	Yes (%)	14 (13.5)
COP	Yes (%)	104 (100)
A-DROP score	0/1/2 (%)	40/44/20 (38.5/42.3/19.2)
Severity	Mild/moderate (%)	40/64 (38.5/61.5)
A-DROP: age (males over 70 years old and females over 75 years old)	Yes (%)	60 (57.7)
A-DROP: dehydration (physical examination or BUN over 21 mg/dL)	Yes (%)	17 (16.4)
A-DROP: respiration (SpO_2_ less than 90% or PaO_2_ less than 60 Torr)	Yes (%)	7 (6.7)
A-DROP: orientation (disturbance of consciousness)	Yes (%)	0 (0)
A-DROP: pressure (systolic blood pressure less than 90 mmHg)	Yes (%)	0 (0)
Admission	Yes (%)	88 (84.6)
Pleural effusion on chest X-ray	Yes (%)	5 (4.8)
Presence of comorbidities	Yes (%)	72 (69.2)
Days of intravenous infusion	2/3/4 (%)	70/29/5 (67.3/27.9/4.8)
Days of oral medication	2/3/4/5/ greater than or equal to 6 (%)	1/6/25/54/18 (1.0/5.8/24.0/51.9/17.3)

The median (interquartile range) age of the patients was 72 years (range: 62-79 years). According to the A-DROP scoring system, 40 (38.5%) and 64 (61.5%) cases were classified as mild and moderate, respectively. To enroll patients with COP, 14 cases (13.5%) of NHCAP and 90 cases (86.5%) of CAP were included. Seventy-two patients (69.2%) possessed comorbidities. Finally, the duration of the LSFX infusion treatments was three days or less in 99 (95%) of 104 cases, and that of the LSFX tablet therapies was five days or less in 86 (82.8%) of 104 cases (Table 1).

The presumed pneumonia-causing organisms are listed in Table 2.

**Table 2:** Causative bacteria (SAS) MSSA: methicillin-susceptible *Staphylococcus aureus*; SAS: safety analysis set

Causative bacteria	Cases (%)
Oral *Streptococcus* spp.	62 (52.0)
Haemophilus influenzae	17 (14.0)
Moraxella catarrhalis	13 (11.0)
*Prevotella* spp.	12 (10.0)
*Actinomyces* spp.	11 (9.2)
*Neisseria* spp.	11 (9.2)
*Rothia* spp.	7 (5.8)
*Veillonella* spp.	6 (5.0)
Chlamydia pneumoniae	5 (4.2)
Streptococcus pneumoniae	4 (3.3)
*Fusobacterium* spp.	4 (3.3)
Legionella pneumophila	3 (2.5)
*Staphylococcus aureus *(MSSA)	1 (0.8)
Gemella haemolysans	1 (0.8)
Parvimonas micra	1 (0.8)
Pseudomonas aeruginosa	1 (0.8)
Mycoplasma pneumoniae	1 (0.8)
Unclassifiable	6 (5.0)

In patients with SAS, oral *Streptococcus* spp. were the most frequently detected organisms (62 cases (52.0%)), followed by *Haemophilus influenzae* (17 cases (14.0%)) and *Moraxella catarrhalis* (13 cases (11.0%)). However, *Streptococcus pneumoniae* was relatively rare (four cases (3.3%)). Diverse anaerobic bacteria were detected in the remaining cases, with *Prevotella *spp. in the majority (12 cases (10.0%)) of the remaining cases (Table 2). *P. aeruginosa* was detected as the causative organism in only one case (0.8%), suggesting that antimicrobial agents against *P. aeruginosa* as empiric therapy may not be necessary for mild-to-moderate COP.

Table 3 summarizes the clinical efficacy rates of TOC, early clinical efficacy testing, and EOT in the FAS and PPS populations. The primary endpoint, the cure rate at TOC in the FAS population, was 100/104 (96.2%), which was favorable. The secondary endpoints of early clinical response and EOT in the FAS population were 101/104 (97.1%) and 103/104 (99.0%) (Table 3), respectively, and the PPS population showed a high efficacy rate (Table 3).

**Table 3:** Clinical efficacy (FAS and PPS) FAS: full analysis set; PPS: per-protocol analysis set; TOC: test of cure; EOT: end of treatment

FAS	Clinical response (N = 104)	
	Cured or effective (%)	95% confidence interval
TOC	100 (96.2)	90.44-98.94
Early clinical efficacy	101 (97.1)	91.80-99.40
EOT	103 (99.0)	94.76-99.98
PPS	Clinical response (N = 99)	
	Cured or effective (%)	95% confidence interval
TOC	95 (96.0)	89.98-98.89
Early clinical efficacy	96 (97.0)	91.40-99.37
EOT	98 (99.0)	94.50-99.97

The FAS population was stratified by causative organisms (oral *Streptococcus* spp. and anaerobes), age </≥ 75 years, severity according to the A-DROP score, presence of complications, and sensitivity analysis (Table 4).

**Table 4:** Clinical efficacy in subgroup analysis (FAS) FAS: full analysis set; TOC: test of cure; A-DROP: age, dehydration, respiration, disorientation, and blood pressure *The summary of 95 cases is shown because nine of 104 FAS cases were not determined to be causative organisms.

TOC		Clinical response	
		Cured or effective (%)	95% confidence interval
Oral *Streptococcus* spp.*	Yes (N = 52)	51 (98.1)	89.74-99.95
	No (N = 43)	40 (93.0)	80.94-98.54
*Anaerobic* spp.*	Yes (N = 19)	18 (94.7)	73.97-99.87
	No (N = 76)	73 (96.1)	88.89-98.18
Under 65 years old	Yes (N = 31)	29 (93.5)	78.58-99.21
	No (N = 73)	71 (97.3)	90.45-99.67
Under 75 years old	Yes (N = 65)	63 (96.9)	89.32-99.63
	No (N = 39)	37 (94.9)	82.68-99.37
Severity (A-DROP)	Mild (N = 40)	38 (95.0)	83.08-99.39
	Moderate (N = 64)	62 (96.9)	89.16-99.62
Comorbidity	Yes (N = 72)	69 (95.8)	88.30-99.13
	No (N = 32)	31 (96.9)	83.78-99.92

High efficacy rates were observed regardless of the causative microorganisms (oral *Streptococcus* spp. and anaerobes), age (>75 years), disease severity, or the presence of complications. Furthermore, when the sensitivity analysis was performed for patients with NHCAP, chronic kidney disease, and chronic liver disease, the efficacy rates were high in all stratified populations (Table 5).

**Table 5:** Clinical efficacy in subgroup analysis (FAS) FAS: full analysis set; NHCAP: nursing and healthcare-associated pneumonia; TOC: test of cure; EOT: end of treatment

NHCAP	Clinical response (N = 14)			
	Cured or effective (%)			95% confidence interval
TOC	13 (92.9)			66.13-99.82
Early clinical efficacy	12 (85.7)			57.19-98.22
EOT	14 (100.0)			76.84-100.00
Chronic kidney diseases			Clinical response (N = 5)	
			Cured or effective (%)	95% confidence interval
TOC			5 (100.0)	47.82-100.00
Early clinical efficacy			5 (100.0)	47.82-100.00
EOT			5 (100.0)	47.82-100.00
Chronic liver diseases		Clinical response (N = 5)		
		Cured or effective (%)		95% confidence interval
TOC		5 (100.0)		47.82-100.00
Early clinical efficacy		4 (80.0)		28.36-99.49
EOT		5 (100.0)		47.82-100.00

To assess the microbiological efficacy, the eradication rate of microorganisms detected before treatment was assessed for the FAS population, as shown in Table 6.

**Table 6:** Microbiological efficacy (FAS, TOC) FAS: full analysis set; TOC: test of cure

Characteristic	Patient number (%)
Eradication of microorganisms detected before treatment	84 (92.3)
Persistence of microorganisms detected before treatment	7 (7.7)
Missing (no determination of causative organism)	13

To examine the impact of switch therapy on the length of hospital stay, the median (interquartile range) time required for hospitalization to the day of meeting discharge criteria was determined (6.0 days (range: 2.0-7.0 days), which was shorter than the actual median (interquartile range) hospital stay of 7.0 (4.5-8.0) days (Table 7).

**Table 7:** Length of hospitalization (FAS) Missing: no hospitalization for 16 cases and continued hospitalization for 13 cases for actual length of hospitalization; no hospitalization for 16 cases, failure to meet discharge criteria for one case, and discharged but no date confirmed for achieving discharge criteria for five cases for the necessary length of hospitalization CI: confidence interval; FAS: full analysis set

Actual length of hospitalization	Days
Days of hospitalization (median, 95% CI)	7.0 (4.5, 8.0)
The necessary length of hospitalization	Days
Days by fulfillment of discharge criteria (median, 95% CI)	6.0 (2.0, 7.0)

Furthermore, in the FAS population, the exact number of patients discharged at EOT or TOC was substantially lower than those who met the discharge criteria, irrespective of hospitalization or discharge, showing a discrepancy between actual discharge and dischargeable status (Table 8).

**Table 8:** Continued hospitalization with or without fulfillment of discharge criteria (EOT, TOC) TOC: test of cure; EOT: end of treatment

EOT	Yes (%)
Continued hospitalization due to failure to meet the discharge criteria	6 (6.8)
Continued hospitalization despite fulfillment of discharge criteria	36 (41)
Discharge	46 (52)
TOC	Yes (%)
Continued hospitalization due to failure to meet the discharge criteria	1 (1.1)
Continued hospitalization despite fulfillment of discharge criteria	12 (14)
Discharge	75 (85)

All the reasons for the discrepancy in hospitalization were attributed to family circumstances and the wait for transfer to another hospital, rather than medical necessities. Twelve adverse events related to LSFX were observed, and worsening pneumonia was reported as a serious adverse event (Table 9).

**Table 9:** Adverse events (SAS) SAS: safety analysis set *Serious adverse event

Contents	LSFX-related adverse events	
	N	%
Hepatic dysfunction	5	4.2
Hypereosinophilia	1	0.8
Kidney injury	1	0.8
Diarrhea	1	0.8
Loose stool	1	0.8
Worsening of pneumonia*	1	0.8
Drug-induced pneumonia	1	0.8
Phlebitis	1	0.8
Total number of events	12	10.0

## Discussion

Pneumonia is a major cause of death in Japan. Thus, effective therapies are required to improve pneumonia prognosis among patients in Japan. In this study, we showed that switch therapy using LSFX was feasible in approximately 114 (95%) of 120 patients with mild-to-moderate COP, with high efficacy rates: 100/104 (96.2%) at TOC, 101/104 (97.1%) at early clinical response, and 103/104 (99.0%) at EOT. High efficacy rates were also observed in patients aged 75 years or older and in those with comorbidities such as chronic kidney disease, chronic liver disease, and NHCAP, indicating that LSFX switch therapy is highly effective even in elderly patients with comorbidities. In a previous study, we reported that switching from sulbactam/ampicillin (SBT/ABPC) to garenoxacin (GRNX) in patients with mild-to-moderate CAP was associated with a clinical efficacy of 94.4% [16]. Consistently, in this study, we found that switch therapy was associated with a higher efficacy. In a retrospective study of 372 Japanese hospitals, using Diagnosis Procedure Combination (DPC) data, it was found that 30.1% of patients switched from intravenous to oral antibiotic administration; the median (interquartile range (IQR)) duration of intravenous administration was seven days (range: 5-10 days) [15], indicating that switch therapy is not yet well established in clinical practice, as the timing for switching therapy was delayed. In the same study, the most common switch was from intravenous β-lactam administration to oral quinolone administration [15], which increased the risk of adverse effects; switch therapy using the same drug (as in the current study) offers a safer approach. A similar retrospective study of 642 hospitals in the U.S. reported that early switch therapy in CAP was implemented in approximately 6% of patients [11], indicating that even internationally, switch therapy is not well established.

In the present study, 46/88 (52%) of the patients were discharged during EOT. Nevertheless, approximately 82/88 (93%) of the patients could be discharged theoretically according to the discharge criteria, suggesting that it is possible to further reduce the length of hospital stay. Reasons for continuing hospitalization despite meeting the requirements were not obtained in the study; however, social reasons are likely the cause of delayed discharge. Early switch therapy is not associated with worse outcomes but is associated with shorter hospital stays, shorter periods of antimicrobial treatment, and lower hospitalization costs [11]. In a super-aged society such as that in Japan, early discharge with active switch therapy may minimize various disadvantages associated with hospitalization, such as delirium and deterioration of physical and cognitive functions. LSFX has a lower risk of resistance than conventional quinolone drugs and, consequently, may be highly effective in elderly patients with recurrent pneumonia.

In this study, approximately 62/120 (52.0%) of patients had oral *Streptococcus*, which is consistent with data from a previous report of CAP [4] suggesting that this may be the causative COP microbe in Japan, where pneumonia in the elderly is common. In addition, the drug sensitivity test showed the good susceptibility of quinolone agents against typical causative microorganisms, *S. pneumoniae* (4/4; 100%) and *H. influenzae* (18/19; 94.7%) (Appendix E).

Contradictory, only 12/19 (63.2%) of *H. influenzae* isolated from sputum before treatment showed sensitivity to SBT/ABPC, one of the first-line antimicrobials used for COP (data not shown). Considering the data of drug susceptibility testing, LSFX may be more effective than SBT/ABPC against COP due to *H. influenzae*. Similarly, considering the rarity of *P. aeruginosa* as a causative microbe in mild-to-moderate COP, despite poor antibacterial activity against *P. aeruginosa*, LSFX serves as a promising first-line antimicrobial for the treatment of mild-to-moderate COP. Following the recommendation of the New England Journal of Medicine, careful use of antibiotics may be required if there is a history of *P. aeruginosa* isolation from sputum and gram staining of the sputum shows gram-negative rods [18].

The study had some limitations. First, it was a single-arm study with a relatively small number of patients. For LSFX switch therapy to be recognized as the standard of care for COP in the future, randomized controlled tests using the first-line treatment, such as ABPC/SBT and ceftriaxone, with a larger number of patients, may be necessary. Second, this study was conducted only in Japan, where the majority of the population is elderly, and the results need to be interpreted carefully to determine whether they can be adapted to other countries with different age structures and causative organisms. Third, in this study, the indication for admission and the choice of IV therapy as empiric treatment depended on the attending physician's discretion. However, for most mild cases of A-DROP, initial therapy with oral medication might be preferable. Lastly, elderly patients who had repeated aspiration pneumonia before were excluded.

## Conclusions

Our results suggest that LSFX switch therapy may be recommended as a potentially effective strategy for treating mild-to-moderate COP. Switch therapy using LSFX might be effective for patients in the early stages of frailty to prevent the deterioration of activities of daily living by encouraging early discharge. Further studies, including comparisons with other first-choice antimicrobial agents, would be warranted.

## Appendices

Appendix A

**Table 10:** Exclusion criteria Excluded when the patient meets one of the following criteria (i to xxi)

Number	Criteria
i	Patients with a history of hypersensitivity to quinolone antibacterial agents
ii	Patients with QT prolongation
iii	Patients with hypokalemia
iv	Patients receiving Class IA (quinidine, procainamide, etc.) or Class III (amiodarone, sotalol, etc.) antiarrhythmic agents
v	Patients with severe hepatic dysfunction
vi	Women who are pregnant or may be pregnant
vii	Patients with preexisting seizure disorders such as epilepsy
viii	Patients with arrhythmia, such as severe bradycardia, ischemic heart disease, heart failure, or other cardiac diseases
ix	Patients with myasthenia gravis
x	Patients with aortic aneurysm or aortic dissection, a history of aortic aneurysm or aortic dissection, family history, or risk factors (Marfan syndrome, etc.)
xi	Patients with severe or progressive underlying diseases or complications (e.g., diabetic patients with poorly controlled HbA1c > 8.0% with or without treatment, patients with advanced cancer whose prognosis for life is not expected during the observation period, and patients with advanced cancer requiring surgery)
xii	Patients whose white blood cell count is less than 2,000/mm^3^
xiii	Active tuberculosis (including suspicious cases), the following diseases for which pneumonia evaluation is not possible: primary lung cancer, malignant tumors with lung metastasis, eosinophilic pneumonia, interstitial pneumonia, and cystic fibrosis
xiv	Patients with respiratory tract infections caused by pathogens (e.g., acid-fast bacteria, fungi, and viruses) that are unlikely to respond to research drugs
xv	Patients with bronchial obstruction or a history of obstructive pneumonia (but patients with chronic obstructive pulmonary disease are not excluded)
xvi	Patients receiving systemic administration of other antimicrobial agents within 7 days before study drug administration (long-term macrolide administration and prophylaxis with sulfamethoxazole/trimethoprim are not excluded)
xvii	Patients with active hepatitis B or hepatitis C, HIV infection
xviii	Patients with recurrent aspiration pneumonia
xix	Patients with Eastern Cooperative Oncology Group-Performance Status 3 or 4
xx	Patients who are currently participating in clinical trials or other interventional studies
xxi	Patients judged to be unsuitable for participation in this study by the principal investigators and subprincipal investigators

Appendix B

**Table 11:** Definitions for clinical evaluation at the early clinical response and EOT EOT: end of treatment; CRP: C-reactive protein

Efficacy	Requirements
Effective	If the patient meets one below and meets either 2 or 3 and there is no deterioration in any of the other criteria
	1. Improvement of at least one clinical symptom (improvement of fever is mandatory for patients with fever (≥37.0°C) at the time of enrollment, and improvement of fever is considered as improvement if the fever has decreased from the time of enrollment)
	2. Resolution of pneumonia on chest imaging or improvement from the worst imaging
	3. Resolution or improvement of inflammatory findings (no worsening of white blood cell count or CRP, improvement of white blood cell counts to ≤9,000/mm^3^, or reduction of CRP from its highest value)
Not effective	The criteria for “effective” are not met
Indeterminate	Lack of information to make the evaluation above
	If there is a clear reason other than pneumonia for the worsening of temperature, white blood cell count, or CRP

Appendix C

**Table 12:** Definitions for clinical evaluation at TOC EOT: end of treatment; TOC: test of cure; CRP: C-reactive protein

Efficacy	Requirements
Effective	When all the following 1-5 criteria are met
	1. The patient is judged to be “effective” at the time of EOT
	2. No antimicrobial prescriptions are required from the time of EOT to the time of TOC
	3. None of the patient's temperature, white blood cell count, or CRP levels have deteriorated from the time of EOT as requiring antimicrobial prescription
	4. Symptoms continue to improve compared with those at EOT, and antimicrobial prescriptions are judged to be unnecessary
	5. Chest imaging shows further improvement in the image of pneumonia compared to that at EOT
Not effective	1. The criteria for “effective” are not met
	2. Death due to pneumonia
Indeterminate	If any of the following applies
	1. Missing information regarding clinical symptoms and signs (e.g., absence of hospital visits on the evaluation date)
	2. Presence of a definitive reason other than the primary diseases, which causes exacerbation of body temperature, leukocyte count or profile, or CRP level
	3. When improvement in clinical symptoms and inflammatory findings is observed, systemic antimicrobial agents other than pneumonia have been administered by the time of TOC

Appendix D

**Table 13:** Drugs prohibited from concomitant use ACTH: adrenocorticotropic hormone

Number	Drugs prohibited from concomitant use
i	Agents containing aluminum, magnesium, calcium, iron, and zinc (when lascufloxacin tablets are administered)
ii	Phenylacetic acid and propionic acid, non-steroidal anti-inflammatory and analgesic drugs
iii	Rifampicin, phenytoin, carbamazepine
iv	Theophylline, aminophylline hydrate
v	Corticosteroids (oral and injectable)
vi	Thiazide diuretics, loop diuretics, glucocorticosteroids, ACTH, glycyrrhizin preparations (when administered as intravenous lascufloxacin)
vii	Repaglinide

Appendix E

**Table 14:** Drug sensitivity test LSFX: lascufloxacin; MIC: minimum inhibitory concentration

Streptococcus pneumoniae								
MIC (μg/mL)			0.06			0.12		
LSFX, N			2			2		
Haemophilus influenzae								
MIC (μg/mL)	≦0.008	0.03		0.06	0.12		1.0	16
LSFX, N	1	11		4	1		1	1
